# Metabolic and fecal microbial changes in adult fetal growth restricted mice

**DOI:** 10.1038/s41390-023-02869-8

**Published:** 2023-11-07

**Authors:** Stephanie P. Gilley, Miguel A. Zarate, Lijun Zheng, Purevsuren Jambal, Deaunabah N. Yazza, Sree V. Chintapalli, Paul S. MacLean, Clyde J. Wright, Paul J. Rozance, Kartik Shankar

**Affiliations:** 1 Department of Pediatrics, Section of Nutrition, University of Colorado School of Medicine, Aurora, CO USA; 2 Department of Pediatrics, Section of Neonatology, University of Colorado School of Medicine, Aurora, CO USA; 3 Arkansas Children’s Nutrition Center, University of Arkansas for Medical Sciences, Little Rock, AR USA; 4 Department of Pediatrics, University of Arkansas for Medical Sciences, Little Rock, AR USA; 5 Department of Medicine, Division of Endocrinology, Metabolism, and Diabetes, University of Colorado Anschutz Medical Campus, Aurora, CO USA

## Abstract

**Background:**

Fetal growth restriction (FGR) increases risk for development of obesity and type 2 diabetes. Using a mouse model of FGR, we tested whether metabolic outcomes were exacerbated by high-fat diet challenge or associated with fecal microbial taxa.

**Methods:**

FGR was induced by maternal calorie restriction from gestation day 9 to 19. Control and FGR offspring were weaned to control (CON) or 45% fat diet (HFD). At age 16 weeks, offspring underwent intraperitoneal glucose tolerance testing, quantitative MRI body composition assessment, and energy balance studies. Total microbial DNA was used for amplification of the V4 variable region of the 16 S rRNA gene. Multivariable associations between groups and genera abundance were assessed using *MaAsLin2*.

**Results:**

Adult male FGR mice fed HFD gained weight faster and had impaired glucose tolerance compared to control HFD males, without differences among females. Irrespective of weaning diet, adult FGR males had depletion of *Akkermansia*, a mucin-residing genus known to be associated with weight gain and glucose handling. FGR females had diminished *Bifidobacterium*. Metabolic changes in FGR offspring were associated with persistent gut microbial changes.

**Conclusion:**

FGR results in persistent gut microbial dysbiosis that may be a therapeutic target to improve metabolic outcomes.

**Impact:**

- Fetal growth restriction increases risk for metabolic syndrome later in life, especially if followed by rapid postnatal weight gain.
- We report that a high fat diet impacts weight and glucose handling in a mouse model of fetal growth restriction in a sexually dimorphic manner.
- Adult growth-restricted offspring had persistent changes in fecal microbial taxa known to be associated with weight, glucose homeostasis, and bile acid metabolism, particularly *Akkermansia*, *Bilophilia* and *Bifidobacteria*.
- The gut microbiome may represent a therapeutic target to improve long-term metabolic outcomes related to fetal growth restriction.

## Introduction

Fetal growth restriction (FGR), defined as the failure of a fetus to achieve its genetic growth potential, impacts approximately 20% of pregnancies in low- and middle-income countries and 4–10% in the U.S.^1–3^ The underlying cause of impaired fetal growth may be due to maternal malnutrition or chronic disease, placental disorders such as preeclampsia, environmental factors like high altitude or cigarette smoke exposure, or fetal causes including infection or genetic disorders.^2,4^ Later in life, formerly FGR individuals are susceptible to metabolic syndrome including obesity, type 2 diabetes, and hypertension.^2,5,6^

Rapid weight gain after FGR further exacerbates metabolic morbidity. In humans, school-aged children with rapid postnatal weight gain after fetal growth restriction have higher BMI,^7^ higher central and visceral adiposity^8–10^ and decreased lean muscle mass.^9^ In mice, rapid weight gain early in life results in insulin resistance, increased peripheral and central fat mass, adipocyte hypertrophy, and elevated leptin expression.^11,12^ However, inadequate growth in early life is associated with poor neurodevelopmental outcomes^7,13^ making it critical to target sufficient postnatal growth while mitigating the risks of rapid catch-up growth. In an effort to prevent insufficient weight gain, many FGR infants are fed concentrated infant formulas and high fat/high calorie complementary foods, despite the possible long-term health consequences.

There are multiple mechanisms which contribute to impaired metabolic outcomes in FGR into adulthood including epigenetic changes,^6^ higher adiposity and lower lean body mass,^4,14^ fewer pancreatic β cells, and increased hepatic insulin resistance.^15^ In animal models, FGR contributes to central insulin resistance through hypothalamic gene expression,^11,16,17^ a change further exacerbated by weaning to a high fat diet.^16^ Although important, there is currently limited capacity to substantially modify these factors in a clinical setting.

Emerging evidence strongly suggests an important role for the gut microbiome, which can be modified by dietary intake in infancy,^18^ childhood,^19^ and adulthood.^20^ The gut microbiome regulates nutrient absorption, digestion, and energy expenditure^21^ and is known to influence long-term health of the entire body, including cardiovascular health and weight gain/obesity.^22–24^ Disturbances in gut microbial composition (dysbiosis) have been reported in FGR piglets^25^ and rats^26^ and in humans in the first few days of life,^27^ but the role of the microbiome in FGR metabolic outcomes has not yet been explicitly studied, nor has the influence of offspring sex.

In this study, we sought to determine whether a high fat weaning diet impacts growth and metabolic outcomes in a mouse model of FGR. Our studies also examined whether gut microbial ecology was altered in adult mice after FGR and whether any adult outcomes correlated with specific fecal microbial taxa.

## Methods

### Ethical approval

All animal experiments and procedures were approved by the University of Colorado Institutional Animal Care and Use Committee and conducted in compliance with the American Association for Accreditation for Laboratory Animal Care at the Perinatal Research Center at the University of Colorado School of Medicine (Aurora, CO).

### Murine model of fetal growth restriction

The FGR model has been previously published by our group^28^ and is based on a calorie-restricted diet as described.^29^ Briefly, we used timed syngeneic matings in C57BL6/J female mice bred in-house. Food intake (in grams) was quantified daily from gestation day E9 through delivery for pregnant dams with ad libitum access to food. Daily intakes (considered 100% of needs) were established for each gestational day and differed by dam weight.

For the present study, dams had ad libitum access to food during gestation days E0–E8. From gestation day E9 through parturition, pregnant dams continued with ad libitum feeds (control) or calorie restricted diet (FGR) that provided 70% of needs. Food was weighed and provided daily. To verify induction of FGR, a cohort of pregnant dams were euthanized by CO_2_ intoxication followed by cervical dislocation at day E18.5. Pups were dissected from the placenta and amniotic sack and weighed on an electronic scale. After delivery FGR dams were returned to ad libitum feeding for the duration of lactation. Only litters with at least 6 surviving pups were used for long-term study to avoid influences of litter size^12,30,31^ on postnatal weight gain.

### High fat diet

Twenty-one-day-old control and FGR offspring were randomized to receive control diet (CON; 7% fat from corn oil, 3.8 kcal/g; Harlan TD.09283) or high fat, high sucrose Western diet (HFD, 45% calories from fat, 34% w/w of sucrose, 4.7 kcal/g; Harlan TD.08811). There were four groups for each sex: (1) Control mice fed CON; (2) Control mice fed HFD; (3) FGR mice fed CON; and (4) FGR mice fed HFD. Animals were provided food and water ad libitum and weighed weekly.

### Metabolic studies

An intraperitoneal glucose challenge was performed at age 16 weeks as previously described.^32^ In the morning mice were placed in clean cages with ad libitum access to water but no food. After 6 h, fasting glucose was measured with a Nova StatStrip Xpress2 Glucose Meter (Nova Biomedical, Waltham, MA) from the tail vein following tail snip. Mice were administered 2 g/kg of sterile 30% glucose solution intraperitoneally and glucose was measured at 15, 30, 60, 90, 120, and 150 min. Additional blood was collected at the end of the 6-h fast and 15 min after glucose injection. Blood was allowed to clot for at least 20 min on ice, then spun at 2000 × *g* at 4 °C for 10 min to collect serum. Insulin was measured using Rat/Mouse Insulin ELISA Kit (EMD MilliporeSigma, St. Louis, MO) per manufacturer’s instructions. Homeostasis model assessment of insulin resistance (HOMA-IR) was calculated as [fasting insulin concentration (pmol/L) × fasting glucose concentration (mmol/L)] / 22.5.

Body composition was assessed in live mice at age 16–18 weeks by quantitative magnetic resonance (EchoMRI-900; EchoMRI, Houston, TX) in the University of Colorado Nutrition Obesity Research Center Small Animal Energy Balance Lab (NORC-SEBL). Echo MRI uses nuclear magnetic resonance relaxometry to directly measure total body fat and lean mass. The scan lasts 2–3 min and animals are gently restrained in a clear plastic tube without sedation. Percent fat mass was calculated as fat mass (g) / body weight (g).

Calorimetry studies were performed on a subset of animals (*n* = 7–9 per group per sex) for 4–8 days in the NORC-SEBL using the Comprehensive Lab Animal Monitoring System (Columbus Instruments; Columbus, OH) which assesses oxygen consumption, carbon dioxide production, food intake, and activity among other measures. All mice had ad libitum access to water and CON or HFD as appropriate. Measurements, obtained every 18 min, were averaged over the entire day as well as independently for light (14 h) and dark (10 h) periods. Data from female mice were limited to either 4 or 8 days to account for variations associated with the estrous cycle. Data were discarded by the NORC-SEBL team if the measurements appeared in error (e.g., no measured water consumption). Respiratory quotient was calculated as the volume of carbon dioxide produced divided by the volume of oxygen consumed.

After all experiments, mice were euthanized by CO_2_ intoxication followed by cervical dislocation. Liver and pancreas were dissected, and organ weight was adjusted for total body weight.

### Statistical analysis

Statistical analyses and visualizations were performed in R v4.0.5 or GraphPad Prism v9.3.1. Outliers were removed if greater than 1.5 x the interquartile range (IQR). Shapiro–Wilk test was used to assess assumption of normality. Student’s *t*-test (if normal distribution) or Mann–Whitney test (if non-normal distribution) were used to assess differences in litter size and pup weight. Linear regression was used to determine whether growth rates differed between control and FGR offspring from weaning to 16 weeks. Area under the glucose tolerance curve (AUC) was calculated for each individual animal in Graph Pad Prism v9.4.1. Two-way analysis of variance (ANOVA, if normal distribution) or the Kruskal-Wallis test (if non-normal distribution) were used to assess outcome differences between groups based on fetal growth conditions and weaning diet. Females and males were analyzed separately.

#### Fecal microbiome assessment and analysis

Sixteen-week-old animals were placed individually in empty clean cages until one or more fecal pellets were produced. Pellets were stored at −80 °C. The fecal microbiome was assessed as previously described.^33^ Briefly, bacterial DNA was extracted with the DNeasy PowerSoil HTP 96 kit (Qiagen, Redwood City, CA) including a bead-beating step in 96-well PowerBead plates on a TissueLyser II (Qiagen). Genomic DNA was used for amplification of the 16 S rRNA gene V4 variable region using 515 F/806 R primers. Paired-end sequencing of pooled amplicons was performed using an Illumina MiSeq Instrument (Illumina, San Diego, CA).

Data processing was performed using QIIME2. We used the *microbiome* package ‘core’ function to retain taxa with at least 5 counts in 5% of samples. Microbiota counts, sample metadata and taxonomy information were imported using the *phyloseq*^34^ package. Alpha diversity was determined using the *microeco* package^35^ and two-way ANOVA was used to test differences between groups. Beta diversity was assessed using Aitchison distance, Bray–Curtis dissimilarity, and Jaccard dissimilarity. Multidimensional scaling was used to visualize groups for each sex, and statistical difference was tested using PERMANOVA with 999 permutations. Multivariable associations between groups and taxonomic abundance were assessed using the *MaAsLin2* package.^36^ Fetal growth and weaning diet were considered fixed effects, and analyses were adjusted for co-housing. Taxa were agglomerated at the genus level. All P-values were false discovery rate-adjusted (Benjamini–Hochberg, *q*-values) and features with *q* < 0.3 were considered significant (default for *MaAsLin2*). For clarity, all findings passing an un-adjusted *P* < 0.05 are also included in results. The OTU relative abundance is visualized on a log-transformed axis in figures.

We performed unsupervised principal components analysis (PCA) of genus-level taxa and biplot visualization following centered-log ratio transformation using the *microviz* package.^37^ The SILVA database was used to determine predicted bacterial functions of genus-level taxa.^38^ Group differences were determined using the tax4fun function in the *microeco* package. Relationships between microbial taxa abundance and metabolic outcomes were summarized using distance-based redundancy analysis (db-RDA) Bray–Curtis distances. Associations of microbial taxa with phenotypic outcomes were performed using Spearman correlations using the *microeco* cal_cor function. Due to the high number of zeros, particularly in taxa of interest, we used feature modification that preserved ratios between non-zero values using the package *zCompositions* as previously described.^39^ We then used Pearson correlation to assess relationships between taxa abundance and measured outcomes.

## Results

### Maternal calorie restriction induces FGR

**Fig. 1:**
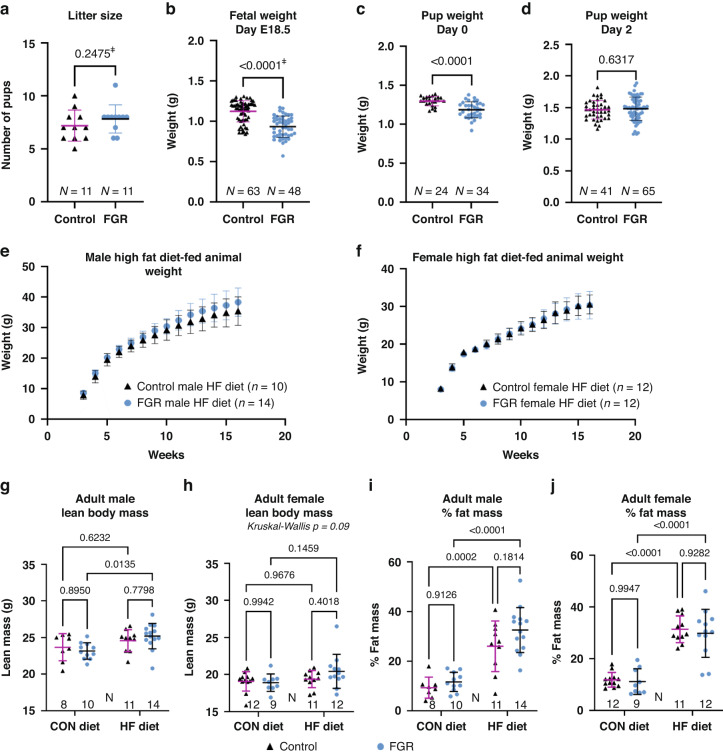
Maternal calorie restriction during pregnancy induces fetal growth restriction. **a** Litter size for control and FGR dams. Offspring weight at (**b**) gestational day E18.5, and day of life (**c**) 0 and (**d**) 2. **e**, **f** Weight (mean ± SD) from 3 to 16 weeks for high fat (HF) diet fed control and FGR (**e**) male and (**f**) female offspring and linear regression analysis of rate of weight gain. **g** Lean body mass in grams for 16-week-old male and (**h**) female offspring. **i** Percent fat mass for 16-week-old male and (**j**) female offspring. ^ǂ^determined by Mann–Whitney test. Bars display mean ± SD.

Calorie restricted dams gained less weight over the second half of gestation (Fig. S1A) without difference in litter size between groups (Fig. 1a). FGR fetuses were on average 17% smaller than control fetuses at gestational day E18.5 (Figs. 1b and S1B). On postnatal days 0 and 1, average weight of FGR pups was less than controls (Figs. 1c and S1D, E) with catch-up growth occurring by postnatal day 2 (Figs. 1d and S1F).

### FGR males gain weight faster on a high fat diet

Control (*n* = 5 litters, 42 pups) and FGR (*n* = 7 litters, 48 pups) offspring were randomized at day of life 21 to control (CON) or high fat (HFD) diet. Body weights at randomization did not differ between female and male offspring, control versus FGR mice, or CON versus HFD groups (Fig. S1G, H). High fat diet resulted in differentiation between CON and HFD fed mice after 3–4 weeks (Fig. S1G, H). Compared with their counterparts fed CON, mice fed HFD weighed more at 16 weeks of age: control males +31.6%, FGR males +38.8%, control females +33.9%, and FGR females +31.5%. Linear regression was used to examine growth rates of HF diet fed animals from 3–7 weeks (from weaning to puberty) and 7–16 weeks (post-pubertal). Male control HFD (slope 1.24, 95% CI: 1.05–1.42) versus FGR HFD mice (slope 1.50, 95% CI: 1.32–1.64) revealed faster post-pubertal weight gain in FGR males (*p* = 0.03; Fig. 1e), a pattern not observed in females (*p* = 0.8; Fig. 1f) or before puberty. Final body weights between control and FGR mice at 16 weeks did not statistically differ for either sex or either diet. At age 16 weeks, lean (Fig. 1g, i) and fat mass were assessed by quantitative MRI. Percent fat mass increased in all HFD groups and was not statistically different between control and FGR mice (Fig. 1h, j).

### FGR males have impaired glucose tolerance on high fat diet

**Fig. 2:**
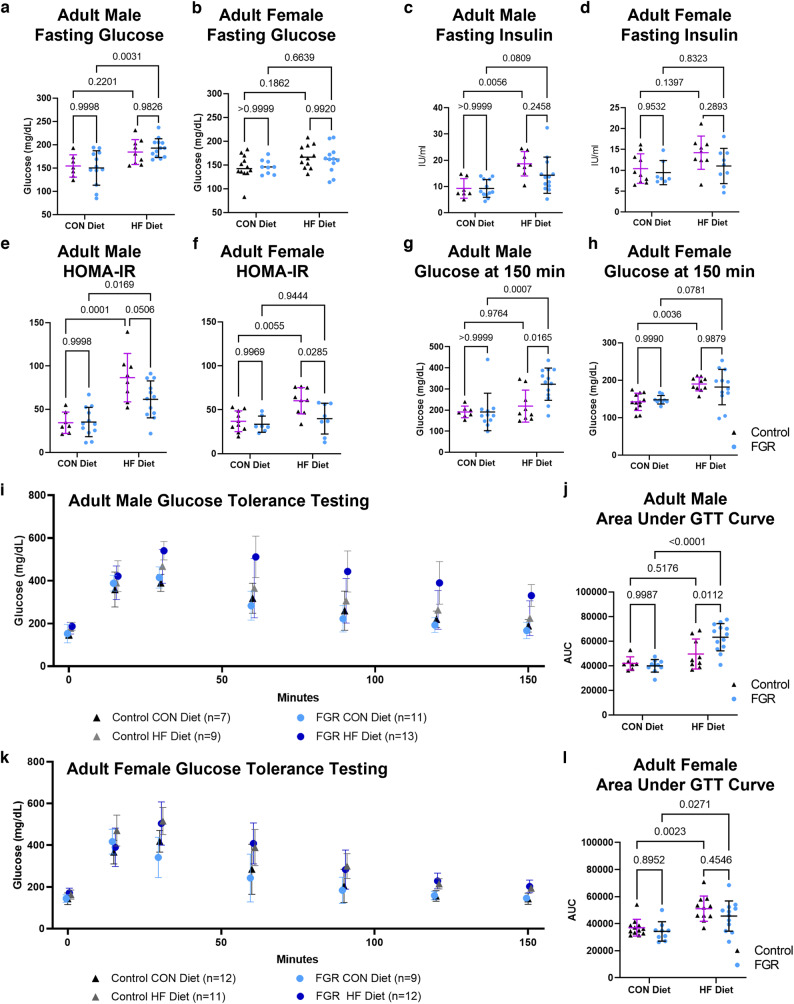
Measures of glucose handling in 16-week-old control and FGR offspring fed control (CON) or high fat (HF) diet. **a**, **b** Mean ± SD serum glucose after 6-h morning fast in male and female mice. **c**, **d** Mean ± SD serum insulin after 6-h morning fast in male and female mice. **e**, **f** Mean ± SD homeostasis model assessment of insulin resistance (HOMA-IR) for male and female mice. **g**, **h** Mean ± SD serum glucose in male and female mice 150 min after 2 g/kg intraperitoneal glucose dose. **i** Mean ± SD serum glucose in male mice during intraperitoneal glucose tolerance test (IP-GTT). **j** Area under the curve (AUC) for IP-GTT in male mice. **k** Mean ± SD serum glucose in female mice during IP-GTT. **l** AUC for IP-GTT in female mice.

Intraperitoneal glucose tolerance testing was conducted after a 6-h morning fast at age 16–17 weeks (*n* = 7–13 per sex per group). Fasting glucose and insulin were impacted by diet but not growth (Table S1). FGR HFD males had higher fasting glucose compared to FGR CON males (Fig. 2a, b). Fasting insulin was higher in control HFD compared to control CON males, but did not otherwise differ based on in utero growth or weaning diet (Fig. 2c, d). Homeostasis model assessment of insulin resistance (HOMA-IR) was calculated from fasting insulin and fasting glucose. Two-way ANOVA showed an effect of diet in both sexes and an effect of intrauterine growth in females only (Table S1). HOMA-IR was higher in control HFD females compared to control CON females without statistical differences between FGR female diet groups (Fig. 2e, f). Both control and FGR males had higher HOMA-IR in the HFD groups; differences between control and FGR HFD groups did not reach significance. HFD resulted in prolonged glucose elevation in control females and FGR males only. At 150 min FGR males fed HFD had significantly higher glucose levels compared to control HFD males (Fig. 2g, h and Table S1). AUC was calculated for each individual mouse then grouped by sex, growth, and diet (Fig. 2i, k and Table S1). In males, FGR but not controls had higher AUC when challenged with HFD (Fig. 2j and Table S1). Both control and FGR females fed HFD had higher AUC compared to CON diet counterparts (Fig. 2l). Diet was a significant driver of AUC in males with an interaction between diet and growth, but intrauterine growth did not reach significance (Table S1). Diet was also a significant driver of AUC in females but the effect of in utero growth was not (Table S1). Liver and pancreas weights as a percent of body weight did not differ based on intrauterine growth (Fig. S2).

### FGR females have lower resting energy expenditure and respiratory quotient

A total of 37 females and 37 males (*n* = 8–10 per sex per group) had successful calorimetry studies. Compared to respective CON fed animals, animals fed HF diet had higher average daily calorie intake, higher resting energy expenditure, and higher total energy expenditure. None of these measures differed based on in utero growth (Fig. S3A–F). Total activity levels (including both moving around the cage and stationary movements such as grooming) did not differ between groups (Fig. S3G, H). Energy balance, defined as energy consumed minus energy expended, was 2.8 to 3.3-fold higher in all HFD groups compared to respective CON group, but did not differ between control and FGR animals (Fig. S3I, J). All HFD groups had lower respiratory quotient compared to respective CON group (Fig. S3K, L). FGR CON females had lower respiratory quotient compared to control CON females (Fig. S3L).

### Microbiome

**Fig. 3:**
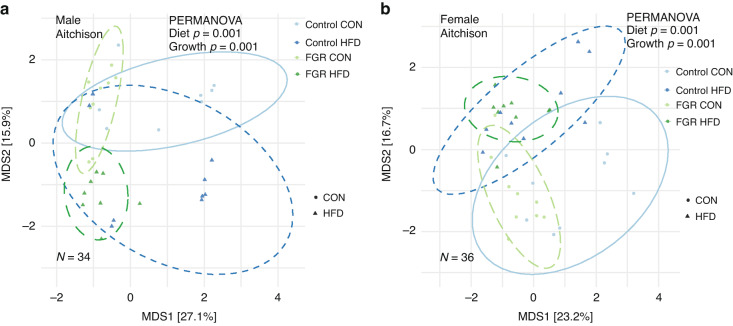
Beta diversity in control and FGR offspring fed control (CON) and high fat (HFD) diet. Aitchison distance differences in (**a**) male and (**b**) female control and FGR offspring.

Alpha diversity was assessed using the *microeco* package and tested for differences using two-way ANOVA. Neither intrauterine growth nor weaning diet altered alpha diversity in adulthood (Table 1). Beta diversity was assessed by Jaccard and Bray–Curtis dissimilarity, as well as Aitchison distance, which may better avoid compositionality bias.^40^ Beta diversity differed in males for both diet and intrauterine growth as assessed in all three methods (Fig. 3a and Table 2). In females, beta diversity differed by intrauterine growth oly based on Aitchison distance only (Fig. 3b and Table 2).

**Table 1:** Alpha diversity measures for fecal microbiome from male and female control and fetal growth restricted (FGR) mice fed control (CON) or high fat (HF) diets.

	Male						Female					
	Control		FGR		*P*-values		Control		FGR		*P*-values	
	CON Diet	HF Diet	CON Diet	HF Diet	Growth	Diet	CON Diet	HF Diet	CON Diet	HF Diet	Growth	Diet
Chao1	27.00 (2.93)	24.78 (2.22)	26.56 (2.30)	26.43 (2.51)	0.494	0.188	27.00 (3.61)	28.67 (1.80)	27.44 (2.46)	26.67 (2.40)	0.385	0.618
Shannon	1.92 (0.30)	1.89 (0.26)	1.77 (0.23)	1.75 (0.17)	0.099	0.770	1.97 (0.15)	2.1 (0.17)	1.97 (0.15)	2.02 (0.19)	0.447	0.095
Simpson	0.80 (0.05)	0.78 (0.07)	0.73 (0.08)	0.69 (0.10)	0.288	**0.010**	0.79 (0.01)	0.83 (0.03)	0.78 (0.07)	0.81 (0.04)	0.441	0.085
Fisher	2.70 (0.23)	2.53 (0.25)	2.68 (0.26)	2.83 (0.27)	0.120	0.930	2.89 (0.17)	2.98 (0.25)	2.87 (0.23)	2.81 (0.25)	0.219	0.814

	*P*-values		
	Both Sexes CON Diet Only		
	Sex	Growth	Interaction
Chao1	0.650	>0.999	0.650
Shannon	0.145	0.344	0.395
Simpson	0.350	0.136	0.218
Fisher	**0.020**	0.799	0.977

Values are presented as mean (SD). *P*-values determined by two-way ANOVA. Bold indicates *P* < 0.05

**Table 2:** Beta diversity measures for fecal microbiome from male and female control and fetal growth restricted (FGR) mice.

	Males		Females	
	Diet	Growth	Diet	Growth
Jaccard	**0.001**	**0.010**	**0.002**	0.067
Bray–Curtis	**0.001**	**0.015**	**0.001**	0.071
Aitchison	**0.001**	**0.001**	**0.001**	**0.001**

Statistical difference was tested using PERMANOVA with 999 permutations. Bold indicates *P* < 0.05.

**Fig. 4:**
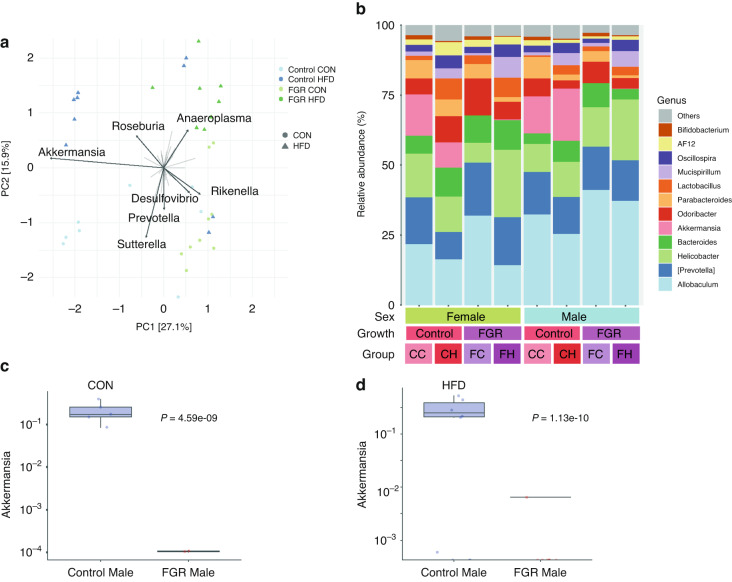
Microbiome taxa in adult control and FGR mice. **a** Unsupervised principal components analysis for male control and FGR mice for both diet groups. **b** Relative abundance of the top 12 genera for all experimental groups at 16 weeks. CC control growth control diet, CH control growth high fat diet; FC FGR control diet, FH FGR high fat diet. *Akkermansia* relative abundance in control and FGR males fed (**c**) control (CON) or (**d**) high fat (HFD) diet (*n* = 8–9 for all groups).

Unsupervised PCA in male mice identified the genera *Akkermansia* and *Rikenella* as the main distinguishing taxa between control and FGR offspring, and *Sutterella* and *Anaeroplasma* differentiated between diet (Fig. 4a). Using taxa aggregated at the genus level, *MaAsLin2* was used to evaluate for gut microbial differences between control and FGR mice of both sexes at 16 weeks. Relative *Akkermansia* abundance was substantially reduced in 16-week-old FGR males in both diet groups (Fig. 4b–d and Table 3). In CON fed females, adult FGR offspring had decreased abundance of *Bifidobacterium* and increased relative abundance of *Sutterella*, while HFD fed females had increased *Prevotella* and decreased *Bilophila* (Table 4).

**Table 3:** Significantly different genera between the microbiome of adult males fed control (CON; top) and high fat (HF; bottom) diet (*n* = 17 total for each diet group).

Male CON Diet—Control versus FGR at 16 weeks of age									
Phylum	Class	Order	Family	Genus	Coefficient	Std Error	*p*-value	*q*-value	*N* not zero
Decreased Abundance									
Verrucomicrobia	Verrucomicrobiae	Verrucomicrobiales	Verrucomicrobiaceae	Akkermansia	−11.153	0.516	**4.59E-09**	**8.70E-07**	7
Proteobacteria	Deltaproteobacteria	Desulfovibrionales	Desulfovibrionaceae	Bilophila	−6.476	1.919	**0.0082**	**0.223**	11
Firmicutes	Clostridia	Clostridiales	Lachnospiraceae	Blautia	−1.752	0.668	**0.028**	0.410	3
Bacteroidetes	Bacteroidia	Bacteroidales	Rikenellaceae	AF12	−2.422	1.015	**0.041**	0.410	16
Bacteroidetes	Bacteroidia	Bacteroidales	Paraprevotellaceae	Paraprevotella	−2.911	1.230	**0.042**	0.410	13
Increased Abundance									
Bacteroidetes	Bacteroidia	Bacteroidales	Bacteroidaceae	Bacteroides	2.428	0.539	**0.001**	**0.057**	17
Firmicutes	Bacilli	Lactobacillales	Lactobacillaceae	Lactobacillus	2.898	0.819	**0.006**	**0.193**	17
Bacteroidetes	Bacteroidia	Bacteroidales	Rikenellaceae	Rikenella	3.507	1.450	**0.039**	0.410	11
Firmicutes	Clostridia	Clostridiales	Lachnospiraceae	Dorea	3.421	1.419	**0.039**	0.410	8

Male HF Diet—Control versus FGR at 16 weeks of age									
Decreased Abundance									
Verrucomicrobia	Verrucomicrobiae	Verrucomicrobiales	Verrucomicrobiaceae	Akkermansia	−10.612	0.182	**1.13E-10**	**1.22E-08**	7
Bacteroidetes	Bacteroidia	Bacteroidales	Paraprevotellaceae	Paraprevotella	−4.029	1.174	**0.011**	**0.148**	8
Increased Abundance									
Firmicutes	Bacilli	Lactobacillales	Lactobacillaceae	Lactobacillus	1.398	0.501	**0.027**	**0.236**	17
Firmicutes	Clostridia	Clostridiales	Lachnospiraceae	Dorea	2.294	0.845	**0.030**	**0.249**	11
Proteobacteria	Deltaproteobacteria	Desulfovibrionales	Desulfovibrionaceae	Desulfovibrio	4.402	1.710	**0.037**	**0.278**	9

Multivariable associations between groups and taxonomic abundance were assessed using MaAsLin2 package and adjusted for cage group. All findings passing an un-adjusted *P* < 0.05 are included in results. Bold indicates *P* < 0.05.

**Table 4:** Significantly different genera between the microbiome of adult females fed control (CON; top) and high fat (HF; bottom) diet (*n* = 18 total for each diet group).

Female CON Diet Control versus FGR at 16 weeks of age									
Phylum	Class	Order	Family	Genus	Coefficient	Std Error	*p*-value	*q*-value	*N* not zero
Decreased Abundance									
Actinobacteria	Actinobacteria	Bifidobacteriales	Bifidobacteriaceae	Bifidobacterium	−2.722	1.001	**0.024**	**0.201**	18
Increased Abundance									
Proteobacteria	Betaproteobacteria	Burkholderiales	Alcaligenaceae	Sutterella	5.465	1.524	**0.006**	**0.126**	16
Bacteroidetes	Bacteroidia	Bacteroidales	Paraprevotellaceae	Paraprevotella	3.753	1.230	**0.014**	**0.193**	13
Bacteroidetes	Bacteroidia	Bacteroidales	Bacteroidaceae	Bacteroides	2.485	0.938	**0.027**	**0.218**	18

Female HFD Diet Control versus FGR at 16 weeks of age									
Decreased Abundance									
Bacteroidetes	Bacteroidia	Bacteroidales	Rikenellaceae	Alistipes	−5.053	0.700	**4.97E-05**	**0.001**	17
Bacteroidetes	Bacteroidia	Bacteroidales	Porphyromonadaceae	Parabacteroides	−5.138	0.780	**1.00E-04**	**0.002**	18
Bacteroidetes	Bacteroidia	Bacteroidales	Paraprevotellaceae	Paraprevotella	−6.403	1.399	**0.001**	**0.020**	15
Proteobacteria	Deltaproteobacteria	Desulfovibrionales	Desulfovibrionaceae	Bilophila	−4.613	1.543	**0.015**	**0.122**	16
Firmicutes	Bacilli	Lactobacillales	Lactobacillaceae	Lactobacillus	−4.106	1.409	**0.017**	**0.127**	18
Bacteroidetes	Flavobacteriia	Flavobacteriales	Flavobacteriaceae	Imtechella	−3.471	1.230	**0.020**	**0.130**	18
Firmicutes	Clostridia	Clostridiales	Lachnospiraceae	Roseburia	−5.746	2.061	**0.021**	**0.130**	8
Bacteroidetes	Bacteroidia	Bacteroidales	Rikenellaceae	AF12	−2.435	0.915	**0.026**	**0.136**	18
Bacteroidetes	Bacteroidia	Bacteroidales	Bacteroidaceae	Bacteroides	−1.898	0.945	0.076	**0.268**	18
Firmicutes	Clostridia	Clostridiales	Christensenellaceae	Christensenella	−3.263	1.707	0.088	**0.291**	9
Increased Abundance									
Bacteroidetes	Bacteroidia	Bacteroidales	Paraprevotellaceae	Prevotella	7.929	1.173	**8.29E-05**	**0.002**	17
Tenericutes	Mollicutes	Anaeroplasmatales	Anaeroplasmataceae	Anaeroplasma	4.453	0.686	**1.13E-04**	**0.002**	4
Bacteroidetes	Bacteroidia	Bacteroidales	Rikenellaceae	Rikenella	2.581	1.106	**0.044**	**0.194**	12
Firmicutes	Clostridia	Clostridiales	Lachnospiraceae	Dorea	3.965	1.823	0.058	**0.237**	12

Multivariable associations between groups and taxonomic abundance were assessed using MaAsLin2 package and adjusted for cage group. All findings passing an un-adjusted *P* < 0.05 are included in results. Bold indicates *P* < 0.05.

**Fig. 5:**
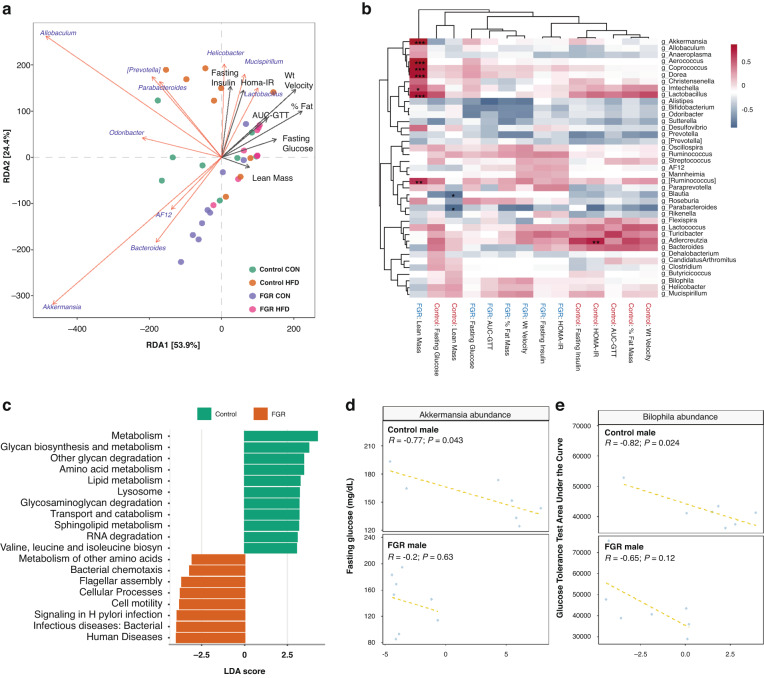
Association of genus-level taxonomic abundance with metabolic outcomes. **a** Distance-based redundancy analysis for adult control and FGR males. **b** Hierarchical clustering of correlations between genera and phenotypic outcomes. Red-blue spectrum denotes positive to negative correlations, respectively. *p < 0.05, **p < 0.01, ***p < 0.001. **c** Predicted microbial functions in control and FGR males. **d** Association between *Akkermansia* abundance and fasting glucose in 16-week-old control (top) and FGR (bottom) males fed control diet. **e** Association between *Bilophila* abundance and area under the glucose tolerance curve in 16-week-old control (top) and FGR (bottom) males fed control diet.

We next asked whether abundance of any taxa was associated with measured metabolic outcomes including weight gain velocity, percent fat mass, fasting glucose, HOMA-IR and GTT AUC. We focused on males due to the presence of greater microbial and metabolomic differences. In adult males, dbRDA of genus-level taxonomic abundance and outcomes showed negative alignment of *Akkermansia* and metabolic outcomes, particularly velocity of weight gain, % fat mass, GTT AUC and HOMA-IR (Fig. 5a). In CON-fed FGR males, there was a strong positive correlation between lean mass and relative abundance of *Akkermansia*, *Ruminococcus*, and other genera (Fig. 5b). Predicted bacterial functions showed differences between control and FGR males including metabolic functions (Fig. 5c). Evaluation of relationships between zero-adjusted taxonomic abundance of *Akkermansia* and *Bilophila* with outcomes revealed associations in control CON males only: higher *Akkermansia* abundance was associated with lower fasting glucose (Fig. 5d), and higher *Bilophila* abundance was associated with lower GTT AUC (Fig. 5e). These associations were not significant in females, FGR males, or HFD-fed control males.

## Discussion

Fetal nutrient insufficiency results in developmental programming changes to prepare for postnatal nutrient scarcity.^6^ Although typically adaptive in utero, these changes are hypothesized to predispose to metabolic impairments such as obesity, type 2 diabetes, and cardiovascular disease in the setting of postnatal nutrient abundance.^41^ The long-term metabolic consequences of fetal growth restriction are well documented^2^ as are the risks of subsequent rapid postnatal growth.^10^ Little research has focused on the effect of high fat diet consumption later in life or on a possible role of gut microbial dysbiosis. In this study, we report adverse effects of high fat diet challenge following FGR and persistent gut microbiome changes into adulthood. In particular, our results demonstrate faster weight gain, glucose intolerance, and reduced relative abundance of *Akkermansia* in male mice with a history of FGR.

Among males, FGR mice fed HFD gained weight faster than control HFD mice, despite equivalent energy balance. Prior work in mice reported similar findings, although only studied male offspring.^12,42^ In one study, FGR males fed high calorie diet had distinctly higher weight compared to control animals starting around age 4 months, even though energy intake did not differ.^12^ At our study’s conclusion, weight was not statistically different between CON-fed control and FGR animals of either sex; however, endpoints were obtained earlier than existing studies (16 weeks versus 6-9 months), raising the possibility that extension to older ages may further distinguish our two groups.

We also report impaired glucose handling in FGR HFD males compared to control HFD males, without differences in females. Prior work, including in a model similar to ours,^43^ also showed impaired glucose handling in FGR males at 6 to 9 months of age,^12,43^ though there was no consideration of sex as a biologic variable. Sex differences in response to HFD are well documented in mice, generally demonstrating relative protection in females.^44,45^ In this cohort, control males fed HFD had higher fasting insulin and higher HOMA-IR compared to CON-fed control males, but IP-GTT testing did not differ. In contrast, FGR males showed glucose intolerance in response to HF diet, indicating higher sensitivity to dietary challenge. In addition, development of impaired glucose handling prior to weight divergence between the two groups suggests that glucose intolerance is an early phenomenon in FGR and may drive cardiovascular disease and excess weight gain. We did not see statistical differences in serum insulin levels 15 min after glucose injection, suggesting against impaired insulin secretion. However, to more deeply understand the etiology of glucose intolerance, future work can include targeted studies of insulin signaling including insulin tolerance testing and/or metabolic clamp studies.^11,46^

Sexual dimorphism has been reported previously in multiple FGR animal models as well as in humans.^47^ Maternal calorie restriction in mice caused sex differences in the offspring’s response to lipopolysaccharide injection.^28^ In a hypoxia-induced FGR mouse model, adult female mice had airway hyper-responsiveness compared to hypo-responsiveness in adult males.^48^ In a rat FGR model, white adipose tissue progenitor cells from males had higher expression of genes related to adipocyte differentiation.^49^ Growth-restricted male guinea pigs had a more adverse response to a high fat diet.^50^ Sexual dimorphism has also been noted in pig^17^ and non-human primate^51,52^ models of FGR. In humans, fetuses respond to intrauterine exposures in a sexually dimorphic manner, which may impact postnatal outcomes.^47^ Male infants have well-documented inferior neonatal morbidity and mortality compared to females, including worse survival and higher rates of serious complications such as necrotizing enterocolitis.^53^

Although sex hormones^49^ and epigenetic changes^6^ may partially explain differences related to intrauterine growth, the role of the intestinal microbiome has been understudied. Fecal microbial taxa have been shown by multiple groups to be associated with feeding tolerance^54,55^ and growth^56,57^ during infancy. The microbial relationship with growth and feeding is particularly critical for FGR neonates who are more likely to receive antibiotics^58^ or to have enteral feeds delayed or interrupted due to feeding intolerance^59^ or concern for necrotizing enterocolitis.^60^ Recent work found an association between neurodevelopment and early fecal microbiota in infants with FGR.^27^ Gut microbial composition could, therefore, have life-long effects on the growth and development of FGR infants and is a logical target for intervention.

Disturbances in gut microbial composition have been reported in FGR piglets,^25,61^ rats,^26^ and in human neonates.^27^ In our mouse model, FGR resulted in persistent fecal microbial changes into adulthood. In particular, FGR males had substantial reduction in relative abundance of *Akkermansia*, a mucin-residing genus known to be associated with intestinal barrier integrity, glucose handling, and weight gain in mice^62–64^ and humans.^65–68^ *Akkermansia* supplementation in mice fed a high sugar diet resulted in improved glucose control and insulin secretion.^62^ In human subjects with overweight and obesity, *Akkermansia* improved insulin sensitivity, weight, adiposity, systemic inflammation, and serum lipopolysaccharide levels, a marker of intestinal barrier strength.^69^ There are multiple proposed mechanisms for the systemic effects of *Akkermansia*,^67^ many of which are impaired in the setting of FGR. *Akkermansia* improves intestinal barrier function^69^ known to be altered in FGR models;^70–72^ increases short-chain fatty acid production,^67^ diminished in FGR pigs;^61^ and facilitates bile acid metabolism,^62^ altered in FGR piglets.^73^ We also report positive correlation of lean mass with *Akkermansia* abundance in FGR males. Skeletal muscle mass is a major driver of insulin sensitivity^74^ and known to be reduced across the lifespan in humans with FGR.^4^ Recent work showed improved muscle strength with *Akkermansia* supplementation in a mouse model of muscle atrophy.^75^ Additional studies are needed in the FGR population to determine whether *Akkermansia* depletion may be related to short- and long-term outcomes.

Male FGR offspring also had reduced relative abundance of *Blautia*, an acetic acid producer whose depletion has been associated with type 2 diabetes, obesity and visceral adiposity^76,77^ and is being tested as a possible therapeutic.^78^ We also detected reduced abundance of *Bilophilia*, known to be depleted with defects of bile acid conjugation.^79^ The exact implications of this finding may depend on which specific bacteria are diminished, as species within this genus have been associated with either beneficial,^80^ or detrimental metabolic^81^ or inflammatory^82,83^ effects. Future work is needed to identify bacterial species and function, and what role bile acids or short chain fatty acids may have in health and disease after FGR.

Fecal microbial alterations due to FGR were less pronounced in adult female offspring. Notably, relative abundance of *Akkermansia* did not differ, supporting a possible role of *Akkermansia* in driving male metabolic outcomes. We also report decreased relative abundance of the beneficial bacteria^84^ *Bifidobacterium*, and increased abundance of *Sutterella*, previously shown to have mild pro-inflammatory effects.^85^ Both genera encompass a variety of species, and their role in FGR likely depends on which exact species are present.^84–86^

The presence of persistent gut microbial changes into adulthood suggests that the gut microbiome may be a logical target for intervention to improve outcomes following FGR. Multiple probiotic strains have shown protective effects against necrotizing enterocolitis.^87^ Other groups have shown that targeted additions to a child’s diet can influence health through the microbiome. In term infants, addition of human milk oligosaccharides to infant formula altered the fecal microbiome and decreased incidence of respiratory illnesses and antibiotic use.^18^ In later childhood, malnourished children fed a nutritional supplement targeting the microbiome had greater weight recovery compared to children fed conventional nutritional therapeutic foods.^19^ However, whether gut microbial changes are similarly found in human adults who experienced FGR is not yet known.

Although the present study has many strengths, it has several limitations. We selected a calorie-restriction model for its translatability to maternal undernutrition, a common cause of FGR worldwide.^1^ Alternative models of FGR include isocaloric low protein diet,^11,12^ late-gestation mesenteric uterine artery ligation,^88^ or infusion of a thromboxane A2 analog,^89,90^ although published studies report milder growth restriction compared to our model. Offspring metabolic outcomes are likely to differ based on the specific model used. We studied offspring into early adulthood which may have limited our detection of certain growth and metabolic endpoints. Studying animals until 6–9 months of age may clarify the true extent of FGR’s longstanding effects.^12,43^ While the tests we performed give broad information about glucose handling, future studies with insulin tolerance testing and/or metabolic clamp studies^11,46^ may further characterize the effects of FGR on glycemic control and elucidate underlying mechanisms. Our use of 16 S sequencing did not allow for identification of specific species nor functional changes of bacteria. We also did not investigate the basis for persistent changes in the microbiome, such as the intestinal microenvironment or other host-microbe interactions. Finally, the association between FGR and gut microbial changes does not prove causation. More studies are needed to determine what mechanistic role, if any, the microbiome plays in metabolic outcomes.

In conclusion, weaning to a high fat diet led to adverse outcomes in male FGR mice compared to controls. FGR mice of both sexes had fecal microbial differences into adulthood, in particular depletion of *Akkermansia* in male offspring. The gut microbiome is a modifiable aspect of health which may be contributing to adverse outcomes associated with FGR and should be targeted for future study.

### Supplementary information

Supplementary Information

## Data Availability

The datasets generated during and/or analyzed during the current study are available from the corresponding author on request.
