# Supplementary material for: Metabolic and fecal microbial changes in adult fetal growth restricted mice
Source: Pediatr Res. Author manuscript; Available in PMC 2024 Mar 8. (PMC10899111; doi:10.1038/s41390-023-02869-8)
Supplement: Supplemental Files [file NIHMS1948105-supplement-Supplemental_Files.pdf]

Supplemental Figures

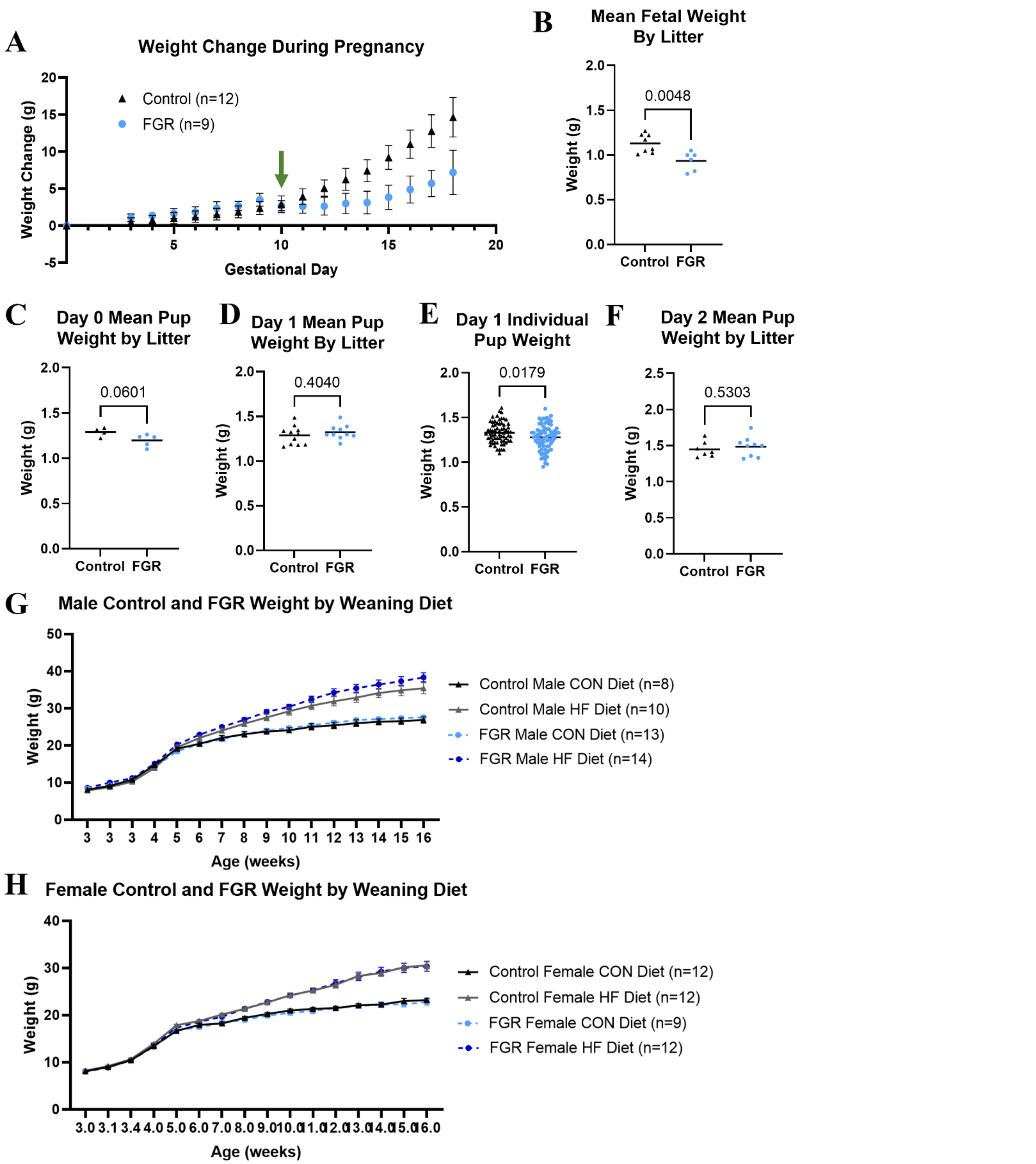

Figure S1: Weight of dams and offspring. (A) Weight gain of pregnant dams in the control (ad lib) versus FGR (calorie restricted) groups. **(B)** Mean fetal weight by litter at gestational day 18.5 and **(C)** day of life 0. **(D)** Mean fetal weight by litter and **(E)** individual fetal weights at day of life 1. **(F)** Mean offspring weight by litter at day of life 2. Offspring weight from age 3 to 16 weeks for **(G)** male and **(H)** female control and FGR offspring fed control (CON) and high fat (HF) diet.

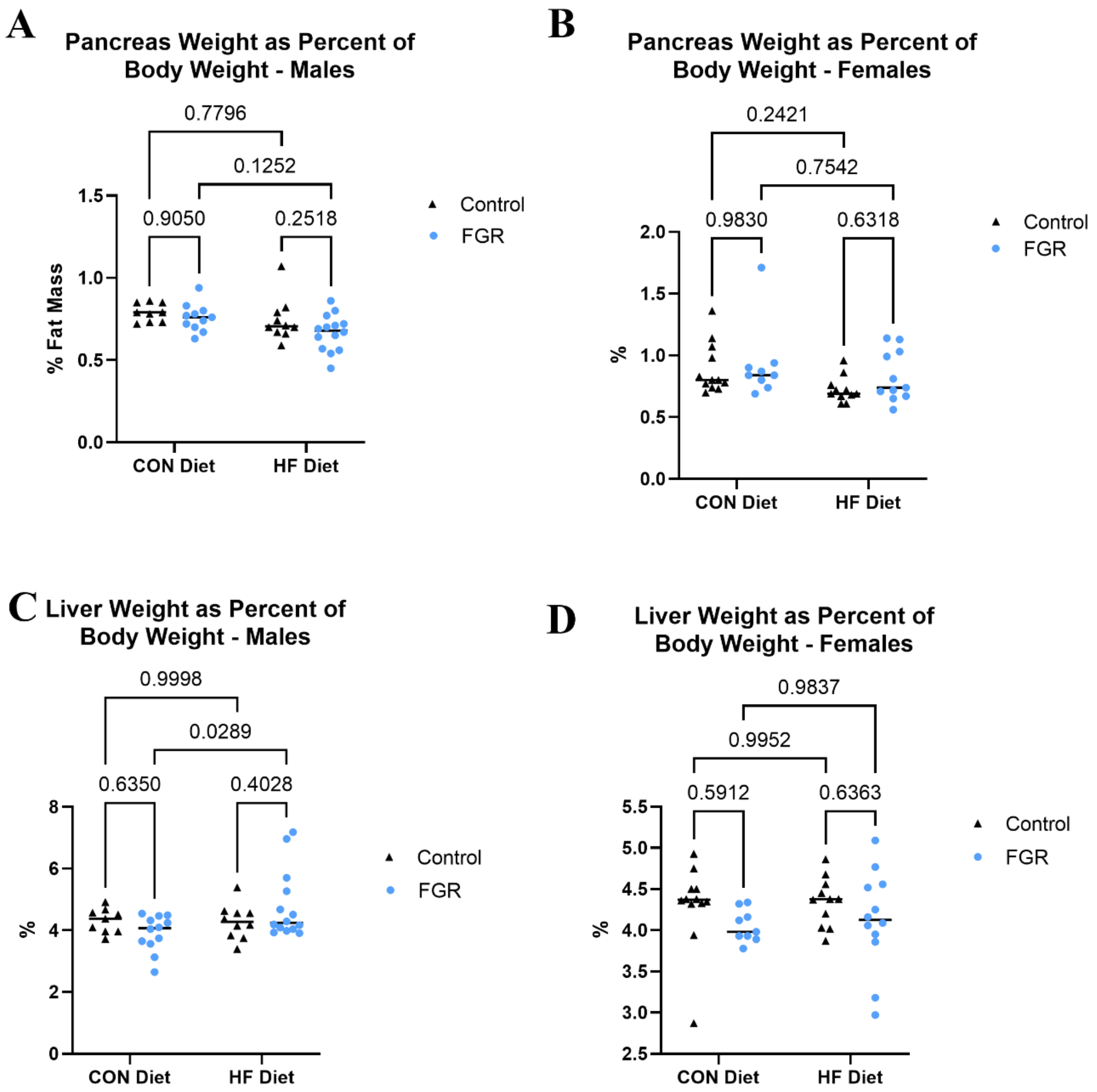

Figure S2: Organ weights in 16-18 week-old control and FGR offspring fed control (CON) or high fat (HF) diet. (A,B) Pancreas weight as percent of body weight in male and female offspring. **(C,D)** Liver weight as percent of body weight in male and female offspring.

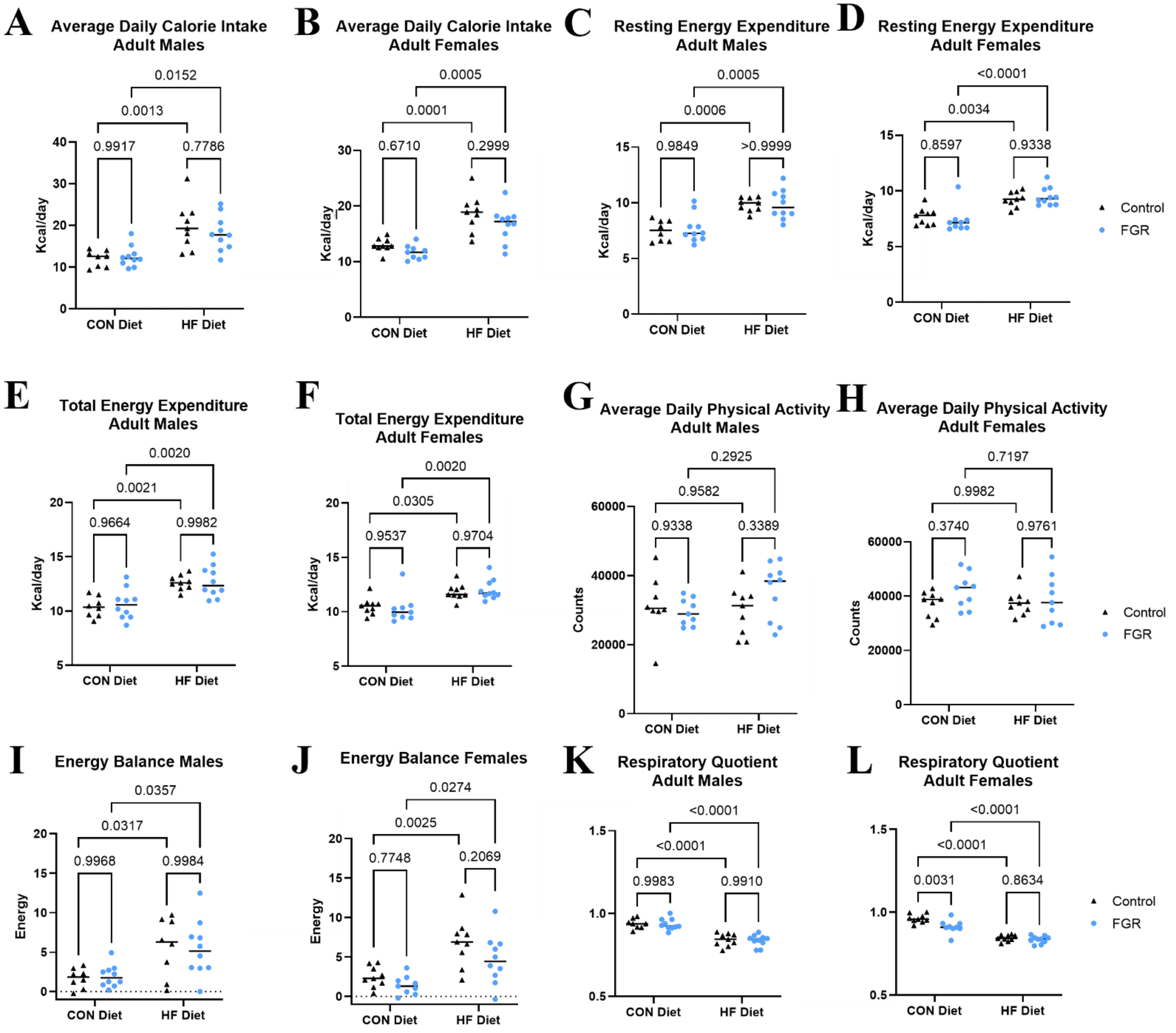

Figure S3: Whole body calorimetry studies for 16-week-old control and FGR offspring fed control (CON) or high fat (HF) diet. (A,B) Mean daily calorie intake for male and female offspring. **(C,D)** Resting energy expenditure for male and female offspring. **(E,F)** Total energy expenditure for male and female offspring. **(G,H)** Mean daily total physical activity for male and female offspring. **(I,J)** Energy balance, calculated as total calorie intake minus total energy expenditure, for male and female offspring. **(K,L)** Respiratory quotient for male and female offspring.

p-values	Males			Females		
	Diet	Growth	Interaction	Diet	Growth	Interaction
Fasting Glucose	**0.0003**	0.818	0.485	**0.016**	0.724	0.650
Fasting Insulin	0.0001	0.199	0.2011	0.042	0.114	0.390
Homeostatic Model Assessment for Insulin Resistance (HOMA-IR)	**<0.0001**	0.080	0.063	**0.004**	**0.018**	0.085
Area Under the Glucose Tolerance Curve	**<0.0001**	0.066	**0.016**	**<0.0001**	0.133	0.612
Glucose at 150 minutes	**0.002**	**0.039**	**0.038**	**<0.0001**	0.875	0.461

Table S1: P-values (ANOVA) for glucose tolerance testing based on weaning diet, intrauterine growth, and the interaction between the two factors.
